# Role of milk and dairy intake in cognitive function in older adults: a systematic review and meta-analysis

**DOI:** 10.1186/s12937-018-0387-1

**Published:** 2018-08-27

**Authors:** Jounghee Lee, Zhuxuan Fu, Mei Chung, Dai-Ja Jang, Hae-Jeung Lee

**Affiliations:** 1 Department of Nutrition Education, Kyonggi University, Suwon-si, South Korea; 2 Department of Epidemiology, University of Pittsburgh, Pittsburgh, USA; 3 Department of Public Health and Community Medicine, School of Medicine, Tufts University, Boston, USA; 4 Research Group of Nutrition and Diet, Korea Food Research Institute, Wanju-gun, South Korea; 5 Department of Food and Nutrition, Gachon University, Seongnam-si, Gyeonggi-do 461-701 South Korea

**Keywords:** Milk, Cognitive decline, Alzheimer’s disease, Meta-analysis, Systematic review

## Abstract

**Background:**

As aging populations increase across the globe, research on lifestyle factors that prevent cognitive decline and dementia is urgently needed. Therefore, a systematic review was conducted to examine the effects of varying levels of milk intake alone or in combination with other dairy products on the outcomes of cognitive function and disorders in adults.

**Methods:**

A comprehensive search was conducted across 3 databases (PUBMED, CINAHL, and EMBASE) from their inception through October 2017. Prospective cohort studies and randomized controlled trials (RCTs) that enrolled adults were included. Studies with follow-up durations of less than 4 weeks and studies including schizophrenic patients were excluded. Two independent investigators conducted abstract and full-text screenings, data extractions, and risk-of-bias (ROB) assessments using validated tools. Studies were synthesized qualitatively using a strength of evidence (SoE) rating tool. A random-effects model for meta-analysis was conducted when at least 3 unique studies reported sufficient quantitative data for the same outcome.

**Results:**

A total of 1 RCT and 7 cohort studies were included. One medium-quality small RCT (*n* = 38 participants) showed that only spatial working memory was marginally better in the high dairy diet group compared to the low dairy diet group. Two of the 7 cohort studies were rated as having a high ROB, and only 1 cohort study was rated as having a low ROB. There were large methodological and clinical heterogeneities, such as the methods used to assess milk or dairy intake and the characteristics of the study populations. It was impossible to conduct a dose-response meta-analysis because the studies utilized different categories of exposures (e.g., different frequencies of milk consumption or the amount of dairy intake). Thus, the overall SoE was rated as insufficient regarding the associations between milk intake and cognitive decline, dementia, and Alzheimer’s disease outcomes. Our meta-analysis of 3 cohort studies showed no significant association between milk intake and cognitive decline outcome (pooled adjusted risk ratio = 1.21; 95% CI: 0.81, 1.82; for highest vs. lowest intake) with large statistical heterogeneity (*I*^*2*^ = 64.1%).

**Conclusions:**

The existing evidence (mostly observational) is too poor to draw a firm conclusion regarding the effect of milk or dairy intake on the risk of cognitive decline or disorders in adults.

**Electronic supplementary material:**

The online version of this article (10.1186/s12937-018-0387-1) contains supplementary material, which is available to authorized users.

## Background

The prevention of cognitive decline and dementia is an increasingly important public health priority due to the growth of the global elderly population [1]. The global prevalence of dementia has been rising, and the number of people with dementia is projected to total 81.1 million by 2040 [2]. Dementia, the most severe cognitive disorder, not only negatively impacts the patients’ quality of life but also creates a substantial burden for caregivers [3, 4].

Cognitive decline is a precursor to mild cognitive impairment (MCI) and is potentially the earliest clinical indicator of dementia [5, 6]. Most patients experience a subjective cognitive decline, also called subjective memory complaint, before noticeable cognitive impairment [7, 8]. Subjective Cognitive Decline (SCD) refers to a self-experienced persistent decline in cognitive abilities in comparison with a prior normal status and independent of the objective performance on neuropsychological tests [9]. By definition, SCD is a sign of preclinical Alzheimer’s disease and can occur before objective cognitive impairment appears. SCD can be diagnosed by several questionnaires of self-reported cognitive performance, such as the Mini Mental Status Examination (MMSE) and the Global Deterioration Scale (GDS) without the use of neuropsychological tests for objective assessment of cognitive function [10]. Currently, there is no ‘gold standard’ measure for SCD [11]. The international SCD Initiative Working Group has systematically identified 34 self-report SCD measures and has found wide variations in the definitions, cognitive domains, optimal items for each domain, item response options, and time frame across measures [12, 13].

SCD is a potential marker for future Mild Cognitive Impairment (MCI). Currently, MCI is diagnosed by one of three criteria: the revised Mayo Clinic Criteria, the Diagnostic and Statistical Manual of Mental Disorders, fifth edition (DSM-5), or the National Institute on Aging-Alzheimer’s Association workgroup (NIA-AA) [14]. Among the three diagnostic criteria, one commonly shared core characteristic is the objective evidence of impairment from standardized neuropsychological tests in ≥1 cognitive domains (i.e., memory, executive function, attention, language, and visuospatial skills) [14]. Although there is no gold standard regarding neuropsychological tests, it is critical to investigate all major cognitive domains for objective cognitive impairment. Dementia [15], including Alzheimer’s disease and vascular dementia, can be detected by objective measures of cognitive impairment and biomarkers, such as β-amyloid (1–42), total tau, and phospho-tau-181 in cerebrospinal fluid [16].

Milk and dairy products are recommended by many dietary guidelines for meeting the daily requirements for calcium, protein, and vitamin B12 intake. These nutrients are important for maintaining good health in older adults. The biological mechanisms linking milk or dairy consumption to cognitive function are not fully understood. It has been postulated that phospholipids in the milk fat globule membrane (MFGM) might affect cognitive function [17]. There are several possible reasons for why the intake of MFGM could benefit cognitive function [18]. First, MFGM contains high levels of choline derivatives (i.e., phosphocholine, glycerophosphocholine, phosphatidylcholine and sphingomyelin) [19]. These compounds may play an important role in the development of the nervous system. Second, sphingomyelin metabolites are essential elements of the myelin sheath that covers the axons of neurons. Therefore, sphingomyelin metabolites support the myelination and production of neurotransmitters in the brain. Additionally, previous studies have suggested that dietary phospholipids are effective transporters of essential fatty acids that could improve brain health by lowering endoplasmic reticulum stress [20], which is known to increase the risk of neurodegenerative disorders such as Alzheimer’s disease. Lastly, the solubility of phospholipids in brain cell membranes may enhance the neuroplasticity of the hippocampus and support dopamine and glutamate transmission [19].

A previous meta-analysis that examined the potential relationship between milk consumption (with or without other dairy products) and cognitive function or disorders showed that the highest level of milk intake compared to the lowest intake level (as defined by the original studies) was significantly associated with a lower risk of cognitive disorders (pooled odds ratio [OR] = 0.72; 95% CI: 0.56–0.93; *I*^*2*^ = 64%) [21]. However, this meta-analysis pooled cohort studies and cross-sectional studies together and did not consider the risk-of-bias across the studies included in the meta-analysis as per the PRISMA (Preferred Reporting Items for Systematic Reviews and Meta-analyses) guideline [22]. A risk-of-bias assessment can help explain variation (heterogeneity) in the results of studies included in a systematic review or meta-analysis, in that more rigorous studies are more likely to yield results that are closer to the truth. The potential limitations of the included studies must be carefully considered in the evidence synthesis in order to obtain reliable conclusions. Therefore, we conducted a systematic review and meta-analysis following the rigorous methods outlined in the Cochrane handbook for systematic reviews to evaluate whether there is a causal relationship between milk intake and cognitive function or dementia.

## Methods

### Identification of studies and study eligibility criteria

The search strategy was developed based on the search strategy used in an earlier meta-analysis by Wu and Sun [21]. The searches were carried out in 3 databases: PubMed (from inception to September 18, 2017), CINAHL (from inception to October 12, 2017) and EMBASE (from inception to October 12, 2017) and were limited to human studies without language restrictions. The complete search strategy is presented in Additional file 1: Table S1. In addition to the citations identified in our searches, all studies included in Wu and Sun’s meta-analysis were also evaluated based on the eligibility criteria of the present study.

For the present systematic review, prospective cohort studies and intervention trials with follow-up durations of longer than 4 weeks in adults, aged 18 years or older, were included. To be included, studies must have compared varying doses of milk intake, alone or in combination with other dairy products (i.e., yogurt and cheese), and reported outcomes related to cognitive function, including any stage of dementia (i.e., cognitive decline, mild cognitive impairments, and dementia) or any type of dementia (i.e., Alzheimer’s disease and vascular dementia). Studies including schizophrenic patients or those that measured only isolated specific nutrients in milk were excluded.

### Study selection process

All citations identified from the literature searches were independently screened by at least two investigators, according to the pre-established screening criteria to exclude irrelevant abstracts (e.g., animal, in vitro, and cross-sectional studies). The abstract screening was performed using an open-source, online software—Abstrackr [23]. Full-text screening was independently executed by two investigators based on the final study eligibility criteria. Conflicts regarding both the abstract and full-text screenings were resolved by group consensus.

### Data extraction, and quality assessment

Two independent investigators extracted data from each included study using the standardized data extraction forms. Discrepancies were resolved between the two investigators. The risk of bias (i.e., quality) of each included study was assessed using validated tools. For prospective cohort studies, we adapted the Newcastle Ottawa Scale (NOS) [24]. The modified NOS tool included quality items regarding potential selection bias, comparability of the comparison groups (e.g., potential for confounding bias), adequate sample size (e.g., power calculation), and potential biases in outcome assessments and selective outcome reporting. The response options for each quality item were high, unclear, or low risk, with detailed instructions on how to reach these judgements (Additional file 1: Table S2). When five or more items were rated as having a high or unclear risk, the overall risk of bias (ROB) was rated as high. When less than two of the quality items were rated as having a high or unclear risk, the overall ROB was rated as low. These cutoffs for rating the overall ROB were arbitrary, as the NOS did not provide guidance for overall ROB rating. For intervention trials, we used the revised Cochrane risk-of-bias tool for the specific intervention trial designs [25]. This ROB tool includes quality items regarding potential bias arising from the randomization process, bias due to deviations from the intended intervention, bias due to missing outcome data, bias in outcome measurement, and bias in selective outcome reporting. The suggested algorithms for reaching ROB judgments, as specified in the tool manual, were followed [25].

### Qualitative synthesis

All the included studies were qualitatively synthesized in narrative form and in summary tables that tabulated the key features of the study populations, study designs, interventions or exposures, outcomes, and results. The strength of evidence (SoE) for major comparisons and outcomes was assessed through a consensus process of the entire research team, using an evidence grading system utilized by the American Diabetes Association and other prominent groups [26, 27]. Briefly, for each outcome, the SoE level was rated as A (Strong), B (Moderate), C (Limited), D (Inadequate), E (Expert Consensus or Clinical Experience), or NA (Not Applicable). Further details can be found in Additional file 1: Table S3.

### Quantitative synthesis

In light of large clinical heterogeneity (e.g., different outcome measures and various milk intake exposures), a random-effects meta-analysis was performed when there were at least three unique studies that reported sufficient quantitative data for the same outcome [28]. After careful examination of all the extracted quantitative data and heterogeneity issues, only prospective cohort studies reporting the associations between milk intake and cognitive decline outcomes could be pooled. Further, a meta-analysis comparing only the highest and the lowest milk intake level was conducted, because data were insufficient to conduct a dose-response meta-regression. Both the Q statistic (considered significant when the *P* value was less than 0.10) and the *I*^*2*^ index were used to quantify the extent of statistical heterogeneity [29]. *I*^*2*^ values of 25%, 50% and 75% were defined as low, moderate and high heterogeneity, respectively. However, these cutoffs are arbitrary and were used for descriptive purposes only [30]. All calculations and meta-analyses were conducted in Stata SE 14 (Stata Corp). The analytical dataset can be found in the Additional file 2. Two-tailed *P* values less than 0.05 were considered significant.

## Results

**Fig. 1:**
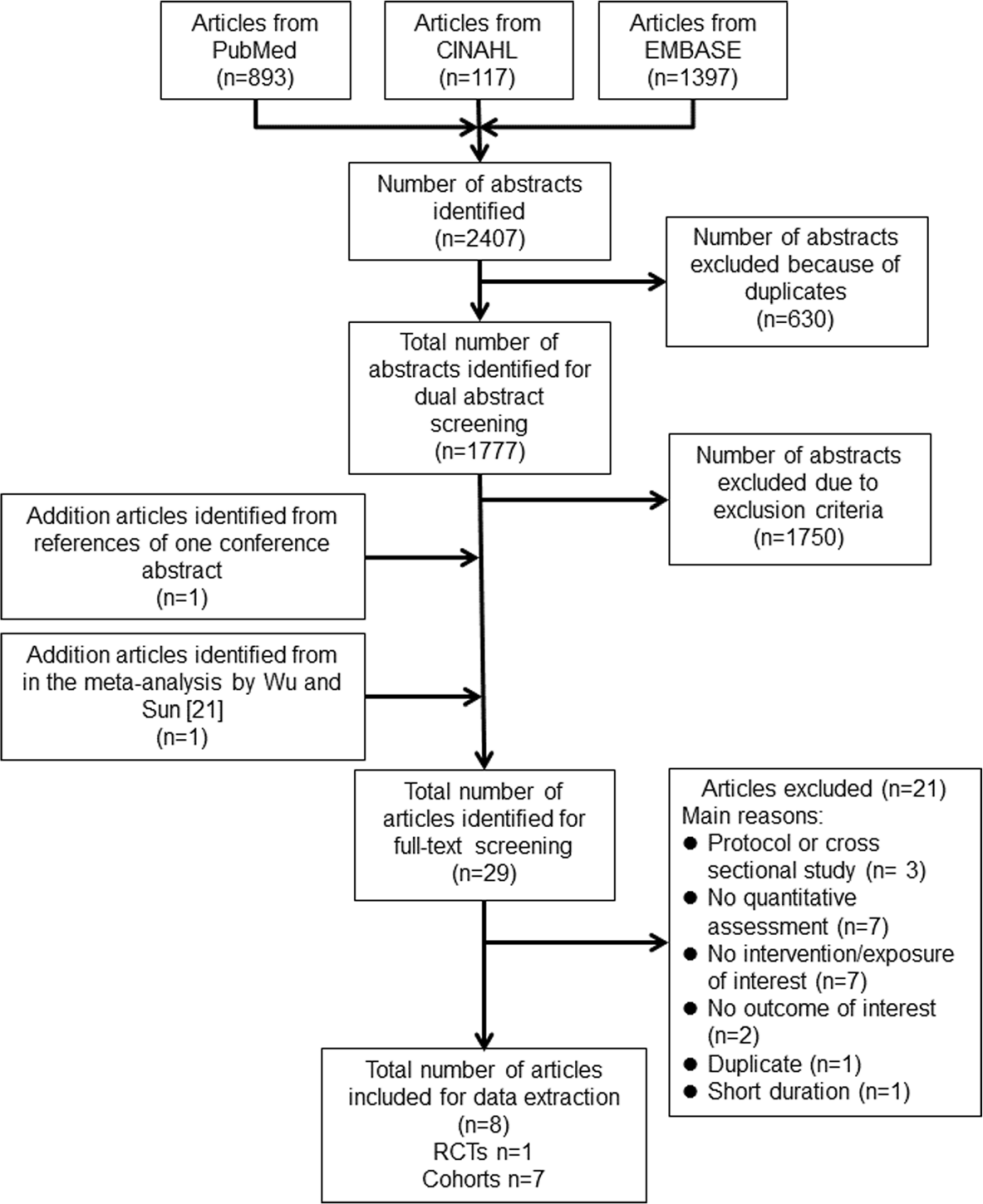
Literature search and study selection process

Our literature search yielded a total of 2407 citations. Of these, 1777 unduplicated abstracts were identified for the dual abstract screening. Further, we screened 29 potentially relevant full-text articles and finally included 8 articles (1 RCT and 7 prospective cohort studies). Figure 1 summarizes the details of the literature search and selection process. The characteristics of the included studies are summarized in Tables 1 and 2. In this paper, we organized results by research questions, namely the effects of milk intake on cognitive function, cognitive decline/impairment, and dementia. One RCT examined only cognitive function outcomes, and the results of this RCT were summarized first, before the results of cohort studies.

**Table 1:** Characteristics of the included RCT studies^a^

Author, Year [Reference]	Country	Duration	Male (%)	Baseline Age (years)	Number of Participants	Intervention	Outcome Measures		ROB assessments
							Type	Method of Assessment	
Crichton, 2012 [31]	South Australia and Canada	6 months	28.9	51.6	38	Four servings of reduce fat dairy/day vs. one serving of reduced fat diary/day	a. Verbal memory b. Processing speed c. Working memory d. Visual attention e. Verbal fluency f. Abstract reasoning g. Selective attention h. Executive function i. Psychological well-being	a. RAVLT total, RAVLT delayed verbal recall, RAVLT written recall b. Digit symbol coding, inspection time c. Spatial Span forward, Spatial Span backward, Spatial Span total, Letter Number Sequencing d. Letter cancellation e. Initial letter fluency f. Matric reasoning g. Stroop interference h. Design fluency total i. DASS total	• Bias arising from the randomization process: Some concerns • Bias due to deviations from intended interventions: Some concerns • Bias due to missing outcome data: High ROB • Bias in measurement of the outcome: Some concerns • Bias in the selection of the reported results: Low ROB • Overall ROB: Moderate (= Some concerns)

^a^ *DASS* The Depression Anxiety Stress Scale, *NA* not applicable, *NR* not reported, *RAVLT* the Rey Auditory Verbal Learning Test

**Table 2:** Characteristics of the included cohort studies^a^

Author, Year [Reference] Cohort name	Country	Follow-Up (years)	Male (%)	Baseline Age (years) (mean)	Health Status	Number of Participants	Exposure			Outcome		Adjustment			
							Type	Method of Easement	Category	Type	Method of Assessment	Nutrients	Demographics	Medication	Life style
Almeida, 2006 [36], NR	Australia	4.8	100	Impaired cognitive function: 77.3; preserved cognitive function: 77.6	Generally healthy	601	Consumed full-cream milk regularly	Self-reported questionnaire	Dichotomous: Regular vs. Rare	Cognitive function	MMSE, GDS-15		X		X
Araki, 2017 [35], Follow up for the J-EDIT trial	Japan	6	42.62	NR	Diabetes mellitus	237	Dairy products	Yoshimura et al.’s validated FFQ	Tertiles of dairy intake (g/d): ≤ 133.9, 134–197.9, ≥ 197	Cognitive function	MMSE		X	X	
Kesse-Guyot, 2016 [32], the SU.VI.MAX 2 observational follow-up study	France	5^b^	52	53.7	Generally healthy	3076	a. Total dairy products; b. Milk only	24-h dietary records	Tertiles of dairy intake (g/d): < 191.6, 191.6–327.2, > 327.2	Cognitive function	TMT, RI-48 test	X	X	X	X
Ozawa, 2014 [38], The Hisayama Study	Japan	17	42.3	Q1: 68.6; Q2: 69.8; Q3: 68.9; Q4: 70.4	Generally healthy	1081	Milk and dairy consumption	a 70-item semi quantitative FFQ	Quintiles (g/d): Male - < 45, 45–96, 97–197, ≥ 198; Female - < 20, 20–75, 76–173, ≥ 174	All-cause Dementia, AD, VaD	DSM	X	X	X	X
Petruski-Ivleva, 2017 [33], The Atherosclerosis Risk in Communities (ARIC) cohort	US	20	44	Milk intake almost never: 56.7; milk intake < 1 glass/day: 57.2; milk intake 1 glass/day: 58.5; milk intake > 1 glass/day: 57.9	Generally healthy	13,752	Milk Intake	FFQ	Almost never, < 1 glass/day, 1 glass/day, > 1 glass/day	Cognitive function	DWRT, DSST, WFT	X	X	X	X
Vercambre, 2009 [34], E3N (Etude Epidémiologique auprès de femmes de la Mutuelle Générale de l’Education Nationale) subcohort	France	13	0	65.5	Generally healthy	4758	Milk and yogurt	an extensive diet history questionnaire covering daily consumption of 208 foods and beverages	Tertiles (no dose for each tertile)	Cognitive decline	DECO score, 4-IADL score	X	X	X	X
Yamada, 2003 [37], Adult Health Study follow-up study	Japan	22^b^	26.8	NR	Generally healthy	1774	Milk intake	self-administered questionnaire between 1965 and 1968	Daily vs. < 4 times a week	AD, VaD	DSM-IV	X	X	X	X

^a^*AD* Alzheimer’s disease, *DECO* observed cognitive deterioration, *DSM* the Diagnostic and Statistical Manual, *DSST* the Digit Symbol Substitution Test, *DWRT* the Delayed Word Recall Test, *FFQ* Food Frequency Questionnaire, *GDS-15* Geriatric Depression Scale 15, *IADL* instrumental activities of daily living, *MMSE* Mini-Mental State Examination, *NR* not reported, *TMT* the Delis-Kaplan trail-making test, *VaD* vascular dementia, *WFT* the Word Fluency Test

^b^Follow-up time is calculated based on the first year of cognitive assessment minus the last year of baseline information assessment, since the data are not reported in the original paper

### Milk and dairy intake and cognitive function

#### RCTs

Only one crossover RCT [31] met the inclusion criteria (Table 1). Participants (*n* = 38) were randomized to either a high-dairy diet (four servings of reduced-fat dairy foods per day) or the low-dairy diet (one serving of reduced-fat dairy foods per day) for 6 months, followed by an alternate diet switch for another 6 months, without a washout period. Nine indicators were used to measure cognitive performance: 1) verbal memory, 2) processing speed, 3) working memory, 4) visual attention, 5) verbal fluency, 6) abstract reasoning, 7) selective reasoning, 8) executive function, and 9) psychological well-being. Among these cognitive function outcomes, only backward spatial span in working memory showed a significant difference between the high-dairy and low-dairy diet groups (mean ± SEM; 7.9 ± 0.4 vs. 7.3 ± 0.4, *P* = 0.046 [31]) (Table 1). The overall ROB of this crossover RCT was rated as moderate due to the high risk for selective outcome reporting bias, some concerns regarding bias due to deviations from intended interventions and bias in outcome measurements, and no information regarding the randomization process (Table 4).

#### Cohort studies

Two cohort studies [32, 33] investigated the associations between milk or dairy intake and cognitive functions among older male and female adults after 5 to 20 years of follow-up in France and in the U.S. (Table 2). One cohort study assessed milk intake using a food-frequency questionnaire (FFQ) [33], and the other evaluated total dairy and milk intake using a 24-h recall [32]. The two cohort studies utilized the following assessment tools to assess cognitive function: DWRT (Delayed Word Recall Test), DSST (Digital Symbol Substitution Test), WFT (Word Fluency Test), RI-48 test, and the Delis-KapLan Trail Making Test. Both studies showed mixed results regarding the associations between milk intake and a variety of cognitive function outcomes. Specifically, the first cohort study showed no significant association between total dairy intake and cognitive function (i.e., working memory and verbal memory [32]). This study indicated no significant association between milk intake and working memory, while a higher milk intake was associated with poorer verbal memory performance [32]. The other study showed no significant association between milk intake and verbal learning, short-term memory, executive function or expressive language but found that a higher milk intake was significantly negatively associated with executive function [33] (Table 3). The overall ROB was rated as moderate for both studies, primarily due to inadequate methods used to ascertain exposure, unclear risk for statistical power and high risk for biased outcome assessment methods (Table 4).

**Table 3:** Results of the prospective cohort studies^a^

Study [Reference]	Outcome	Outcome Definition	Total *N*	No. of Events	Event Rate (%)	Intake	Intake Category	Amount	Metric	Estimate	Lower CI	Upper CI	*P*-value
Kesse-Guyot, 2016 [32]	Working memory	Forward and backward digit span tests and log-transformed TMT score	3076	NA	NA	Total dairy products	Low	< 191.6 g/d	mean difference	ref	ref	ref	0.33
							Medium	191.6–327.2 g/d	mean difference	0.68	−0.16	1.52	
							High	> 327.2 g/d	mean difference	0.43	−0.43	1.29	
	Verbal memory	3 tests including two verbal fluency tasks and RI-48 cued recall task	3076	NA	NA	Total dairy products	Low	< 191.6 g/d	mean difference	ref	ref	ref	0.51
							Medium	191.6–327.2	mean difference	0.05	−0.78	0.89	
							High	> 327.2	mean difference	−0.29	−1.15	0.57	
Petruski-Ivleva, 2017 [33]	Verbal learning and short-term memory	DWRT z	13,752	NA	NA	Milk intake	Almost never	NR	mean difference	ref	ref	ref	NR
							< 1 glass/day	NR	mean difference	−0.04	−0.13	0.06	
							1 glass/day	NR	mean difference	−0.03	−0.14	0.08	
							> 1 glass/day	NR	mean difference	−0.1	−0.2	0	
	Executive function	DSST z	13,752	NA	NA	Milk intake	Almost never	NR	mean difference	ref	ref	ref	NR
							< 1 glass/day	NR	mean difference	−0.04	−0.09	0	
							1 glass/day	NR	mean difference	−0.07	−0.12	− 0.01	
							> 1 glass/day	NR	mean difference	−0.09	−0.14	−0.03	
	Executive function and expressive language	WFT z	13,752	NA	NA	Milk intake	Almost never	NR	mean difference	ref	ref	ref	NR
							< 1 glass/day	NR	mean difference	−0.04	−0.09	0.02	
							1 glass/day	NR	mean difference	−0.02	− 0.08	0.05	
							> 1 glass/day	NR	mean difference	−0.05	−0.11	0.01	
	Cognition	Global z	13,752	NA	NA	Milk intake	Almost never	NR	mean difference	ref	ref	ref	NR
							< 1 glass/day	NR	mean difference	−0.05	−0.11	0.02	
							1 glass/day	NR	mean difference	−0.06	−0.13	0.02	
							> 1 glass/day	NR	mean difference	−0.1	−0.16	−0.03	
Almeida, 2006 [36]	Successful mental health aging	MMSE ≥24 and GDS-15 < 5	601	144	24.0%	Full-cream milk	Rare	NR	HR	ref	ref	ref	NR
							Regularly	NR	HR	0.63	0.45	0.89	
Araki, 2017 [35]	Cognitive decline	Decline in MMSE ≥2	101	33	32.7%	Dairy products	Tertile 1	≤ 133.9	HR	2.8	0.74	10.8	
							Tertile 2	134–196.9	HR	3.6	0.88	15	
							Tertile 3	≥ 197	HR	ref	ref	ref	> 0.05
Vercambre, 2009 [34]	Recent cognitive decline	DECO < 33	4758	518	10.9%	Milk and yogurt	Tertile 1	NR	OR	ref	ref	ref	0.182
							Tertile 2	NR	OR	1.21	0.97	1.5	
							Tertile 3	NR	OR	1.17	0.93	1.46	
	Functional impairment	4-IADL> 0	4758	716	15.0%	Milk and yogurt	Tertile 1	NR	OR	ref	ref	ref	0.799
							Tertile 2	NR	OR	1.04	0.85	1.26	
							Tertile 3	NR	OR	0.97	0.79	1.2	
Yamada, 2003 [37]	Vascular Dementia	DSM-IV	1774	38	2.1%	Milk intake	less than twice a week	NR	OR	ref	ref	ref	
							2–4 times a week	NR	OR	0.418	NR	NR	0.107
							daily	NR	OR	0.257	NR	NR	0.002
	Alzheimer’s Disease	DSM-IV	1774	51	2.9%	Milk intake	less than twice a week	NR	OR	ref	ref	ref	
							2–4 times a week	NR	OR	0.741	NR	NR	0.485
							daily	NR	OR	0.633	NR	NR	0.145
	Vascular Dementia	DSM-IV	1774	38	2.1%	Milk intake	< 4 times a week	NR	OR	ref	ref	ref	0.014
							almost daily	NR	OR	0.35	0.14	0.77	
Ozawa, 2014 [38]	All-cause dementia	DSM-III	1081	303	28.0%	Milk and Dairy Consumption	Quartile 1	women < 45 g/d; men < 20 g/d	HR	ref	ref	ref	0.09
							Quartile 2	women 45–96 g/d; men 20–75 g/d	HR	0.85	0.62	1.18	
							Quartile 3	women 97–197 g/d; men 76–173 g/d	HR	0.69	0.5	0.96	
							Quartile 4	women ≥198 g/d; men ≥174 g/d	HR	0.8	0.57	1.11	
	Alzheimer’s Disease	DSM-III	1081	166	15.4%	Milk and Dairy Consumption	Quartile 1	women < 45 g/d; men < 20 g/d	HR	ref	ref	ref	0.03
							Quartile 2	women 45–96 g/d; men 20–75 g/d	HR	0.64	0.41	0.99	
							Quartile 3	women 97–197 g/d; men 76–173 g/d	HR	0.57	0.37	0.87	
							Quartile 4	women ≥198 g/d; men ≥174 g/d	HR	0.63	0.41	0.98	
	Vascular Dementia	DSM-III	1081	98	9.1%	Milk and Dairy Consumption	Quartile 1	women < 45 g/d; men < 20 g/d	HR	ref	ref	ref	0.14
							Quartile 2	women 45–96 g/d; men 20–75 g/d	HR	1.02	0.59	1.77	
							Quartile 3	women 97–197 g/d; men 76–173 g/d	HR	0.74	0.42	1.33	
							Quartile 4	women ≥198 g/d; men ≥174 g/d	HR	0.69	0.37	1.29	

^a^ *DECO* observed cognitive deterioration, *DSM* the Diagnostic and Statistical Manual, *DSST* the Digit Symbol Substitution Test, *DWRT* the Delayed Word Recall Test, *GDS-15* Geriatric Depression Scale 15, *IADL* instrumental activities of daily living, *MMSE* Mini-Mental State Examination, *NA* not applicable, *NR* not reported, *ref*. reference, *WFT* the Word Fluency Test

**Table 4:** Risk of bias assessment for the included cohort studies^a^

Author, Year	Representativeness of the exposed cohort	Selection of the non-exposed cohort	Ascertainment of the nutrient’s exposure	Outcome of interest absent at baseline	Control for important confounders	Adequate sample size and power	Outcome assessment	Completeness of cohort follow-up	Selective Outcome Reporting	Overall ROB
Almeida, 2006 [36]	High	Low	High	High	Low	Low	High	High	Low	High
Araki, 2017 [35]	High	Low	High	Low	Low	Unclear	High	Low	Low	Moderate
Kesse-Guyot, 2016 [32]	High	Low	Low	High	Low	Unclear	High	Low	Low	Moderate
Ozawa, 2014 [38]	Low	Low	Low	Low	Low	Unclear	Low	Low	Low	Low
Petruski-Ivleva, 2017 [33]	Low	Low	High	Low	Low	Unclear	High	Low	Low	Moderate
Vercambre, 2009 [34]	High	Low	High	High	Low	Unclear	High	High	Low	High
Yamada, 2003 [37]	High	Low	High	High	Low	Unclear	Low	Low	Low	Moderate

^a^Detailed instructions of the modified NOS tool are presented in Additional file 1: Table S2

### Milk and dairy intake and cognitive decline or impairment

**Fig. 2:**
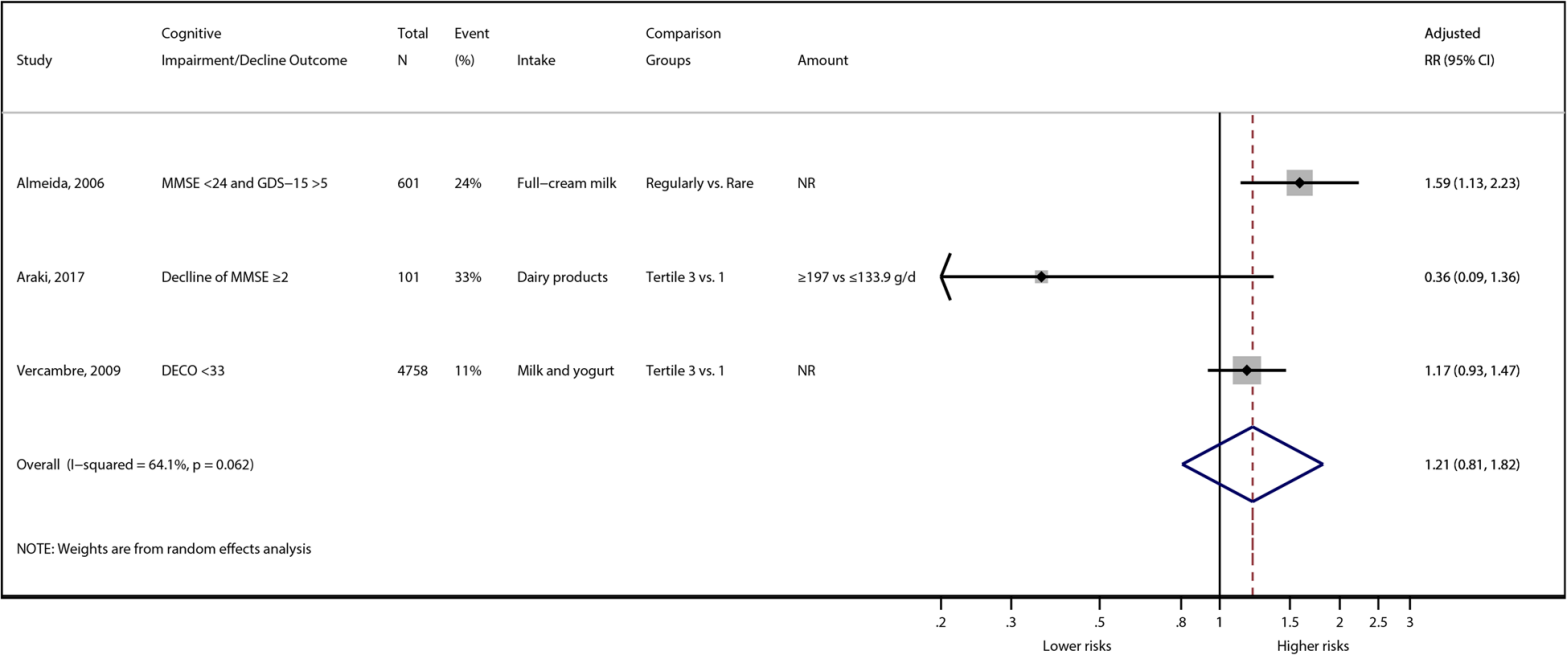
Summary associations between milk intake and cognitive decline

Three cohort studies [34–36] reported the associations between milk or dairy intake and cognitive decline or cognitive impairment among elderly participants after 4.8 to 13 years of follow-up (Table 2). Milk or dairy intake was assessed by FFQ or diet history questionnaire. One of these studies compared a ‘regular’ consumption group with a ‘rare’ consumption group of full-cream milk [36], while the other two studies investigated the associations between cognitive decline/impairments and tertiles of milk intake [34, 35]. Cognitive impairment or decline was assessed using the MMSE (Mini-Mental State Examination), DECO (‘DEtérioration Cognitive Observée’, observed cognitive deterioration) and IADL (Iinstrumental Activities of Daily Living). One cohort study found that the regular full-cream milk consumption group demonstrated a significant decrease in successful mental health aging compared with the rare consumption group (adjusted hazard ratio = 0.63; 95% CI: 0.45, 0.89) [36]. The other two studies found no significant associations between milk and dairy consumption and cognitive decline [34, 35] (Table 3). Our random-effects meta-analysis results did not show significant differences in risk for cognitive decline or cognitive impairment by comparing the highest milk intake to lowest intake groups (pooled adjusted risk ratio = 1.21; 95% CI: 0.81, 1.82), with large statistical heterogeneity (*I*^*2*^ = 64.1%) (Fig. 2). Overall, the ROB was rated as being moderate or high, mainly due to high loss of follow-up (> 20%) and inadequate methods used to ascertain exposure (Table 4).

### Milk and dairy intake and Alzheimer’s disease

Two cohort studies [37, 38] examined the associations between milk or dairy intake and Alzheimer’s disease among elderly participants [38] and adult men and women [37] after 17 and 22 years of follow-up. Milk or dairy intake was assessed using an FFQ, and Alzheimer’s disease was assessed by DSM (Diagnostic and Statistical Manual of Mental Disorders) in both studies (Table 2). The results of these two studies were inconsistent. One study showed that consuming milk less than twice a week was not significantly associated with the risk of developing Alzheimer’s disease, compared with daily or 2 to 4 times a week milk intake [37]. Overall, the ROB of this study was rated as being moderate, primarily due to concerns about the representativeness of the study population and inadequate methods used to ascertain exposure (Table 4). The other study reported that higher milk and dairy intake significantly reduced the risk of Alzheimer’s disease (*P* for trend = 0.03 [38]) (Table 3). Overall, the ROB of this study was rated as being low (Table 4).

### Milk and dairy intake and vascular dementia

Two cohort studies assessed the relationship between milk or dairy intake and vascular dementia, assessed by DSM [37, 38]. The characteristics of these two cohorts were described earlier (Table 2). Similar to Alzheimer’s disease outcomes, these two studies showed inconsistent results for vascular dementia outcomes. Specifically, the first study reported that the almost daily milk intake group (vs. < 4 times a week of milk intake group) had a significantly reduced risk of vascular dementia [37], while the second study showed nonsignificant associations between quartiles of milk and dairy intake and risk of vascular dementia [38]. Overall, the ROB of the two studies was rated as being low and moderate, as described earlier (Table 4).

### Milk and dairy intake and all-cause dementia

One cohort study assessed the association between milk and dairy intake and all-cause dementia, assessed by DSM III, after 17 years of follow-up [38]. The characteristics of this study were described earlier (Table 2). The results showed no significant associations between quartiles of milk and dairy intake and risk for developing all-cause dementia (Table 3). Overall, the ROB of this study was rated as being low (Table 4).

### Overall strength of evidence (SoE)

We graded the overall SoE as being inadequate for the causal relationships between milk consumption and cognitive decline/impairment, all-cause/vascular dementia, and Alzheimer’s disease (Table 5).

**Table 5:** SoE grading: higher vs. lower milk intake by outcome^a^

Outcome	Studies, n (reference)			
	RCTs	Cohort studies	SoE grade	Explanation
Cognitive function	1 [31]	2 [32, 33]	Inadequate	Only 1 RCT was rated as having a moderate ROB; Cohort studies were rated as having a moderate to high ROB
Cognitive decline/impairments	n/a	3 [34–36]	Inadequate	No RCT; Cohort studies were rated as having a moderate to high ROB. Large heterogeneity in the meta-analysis.
All-cause dementia	n/a	1 [38]	Inadequate	Only 1 cohort study that was rated as having a low ROB
Alzheimer’s disease	n/a	2 [37, 38]	Inadequate	Cohort studies that were rated as having a low to moderate ROB showed inconsistent findings
Vascular dementia	n/a	2 [37, 38]	Inadequate	Two cohort studies that were rated as having a low to moderate ROB showed inconsistent findings

^a^The SoE grading scheme is presented in Additional file 1: Table S3

## Discussion

This systematic review revealed that the current evidence (7 cohort studies and 1 RCT) is inadequate to draw a conclusion for the causal relationship between milk or dairy intake and cognitive decline or disorders in older adults. The included cohort studies showed large clinical and methodological heterogeneity, hampering the comparability of the study findings. Specifically, milk or dairy intake assessments were heterogeneous, in that studies used various instruments to measure intake (e.g., food frequency questionnaires [FFQ], 24-h recall, or diet history questionnaires) and definitions of milk intake. The types of milk were also variably defined across studies, such as full-cream milk, dairy products, milk and dairy, or fat-reduced dairy foods. Most cohort studies were conducted in generally healthy populations [32–34, 36–38], except for one [35] that was conducted among individuals with diabetes. Moreover, the included cohort studies utilized a variety of assessment tools to diagnose age-related cognitive disorders and adjusted for different sets of confounding factors in the statistical analyses.

The strength of this systematic review is that we followed the highest methodological standards for evidence synthesis and employed strict inclusion criteria regarding the study designs, which can better determine a causal inference. The previous meta-analysis included cross-sectional studies and did not consider risk-of-bias in their analyses or in drawing their conclusions [21]. Because cross-sectional studies cannot assess the temporality between milk intake and cognitive function, we selected only prospective cohort studies and intervention studies for this systematic review. However, our meta-analysis is limited because of the concerns of risk-of-bias in the included cohort studies (e.g., potential residual confounding) and imprecise measurements of milk intake due to the limitations of FFQs for assessing dietary exposures in observational studies. Additionally, our meta-analysis was based on extreme quantile (highest vs. lowest quintile/quartile) comparisons, which has very limited interpretability, because the definitions of intake levels varied across studies. The variations in exposure levels may explain the large statistical heterogeneity (*I*^*2*^ = 64%; *P* = 0.06) in the meta-analysis, but we could not conduct a dose-response meta-analysis due to insufficient quantitative data to estimate and standardized the ‘doses’ of milk intake levels.

In light of the limitations in this body of evidence, we have a few suggestions for future research. First, the use of biomarkers would overcome the limitations of self-reported dietary assessments. These self-reported dietary assessments are prone to measurement errors due to recall bias, under/over-reporting, and incompleteness of the food composition database [39, 40]. Because self-reported dietary measures rely heavily on the responders’ recall, recall bias is a particularly important issue in the study of cognitive function. Mild cognitive impairment has shown to attenuate the validity of FFQs when comparing to biomarkers of nutrient intake [41, 42]. Poor cognitive ability was associated with suspected recall errors on the FFQ [43]. As an objective complementary tool for dietary assessment, milk intake biomarkers, such as adipose tissue and circulating levels of pentadecanoic acid (C15:0) [44], would be very useful. Second, there is an urgent need to develop validated, standardized tests of cognitive impairment and dementia to make the research outcomes comparable and able to be quantitatively combined. We found substantial heterogeneity in the assessment tools across the included studies and poor descriptions of outcome measures. Each outcome measure for cognitive impairment and dementia exclusively assessed cognition, function, and other domains (e.g., quality of life, mood, and behavior) or a combination of these domains [45]. To increase the usefulness of SCD measures, the international SCD Initiative Working Group issued a call for international collaboration to promote the harmonization and pooling of cognitive self-reported data and greater consistency in the measurement of cognitive decline [12]. The working group had agreed to a common framework and research procedures to study the role of SCD as a marker of preclinical Alzheimer’s disease [9]. Specifically, there is a need to derive a small number of well-constructed, easy-to-administer items with adequate reliability across diverse samples of older adults. In summary, future research could be improved through the use of milk intake biomarkers and standardized assessment tools for cognitive function to strengthen causal inferences.

## Conclusions

Based on best available evidence, we concluded that the overall strength of evidence is inadequate for the effects of milk or dairy consumption on cognitive decline and disorders, due to the insufficient number of high-quality studies and large heterogeneity across studies. To draw a firm conclusion, high quality RCTs with sufficient sample sizes, use of milk intake biomarkers, and standardized assessment tools for cognitive function are needed to identify the role of milk or dairy intake in cognitive function among older adults.

## Additional files

Additional file 1: **Table S1.** Search strategy. **Table S2.** Detailed instructions of the modified NOS tool. **Table S3.** SoE grading system. (DOC 83 kb)

Additional file 2: Analytical dataset. (XLSX 9 kb)

## Abbreviations

DECO: ‘DEtérioration Cognitive Observée’ (observed cognitive deterioration)
DSM: Diagnostic and Statistical Manual of Mental Disorders
DSST: Digital Symbol Substitution Test
DWRT: Delayed Word Recall Test
FFQ: Food frequency questionnaire
GDS: Global Deterioration Scale
IADL: Instrumental Activities of Daily Living
MCI: Mild cognitive impairment
MFGM: Milk fat globule membrane
MMSE: Mini Mental Status Examination
MMSE: Mini-Mental State Examination
NIA-AA: National Institute on Aging-Alzheimer’s Association workgroup
NOS: Newcastle Ottawa Scale
PRISMA: Preferred reporting items for systematic reviews and meta-analyses
RCT: Randomized controlled trial
ROB: Risk of bias
SCD: Subjective Cognitive Decline
SoE: Strength of evidence
WFT: Word Fluency Test
