# Supplementary material for: Role of milk and dairy intake in cognitive function in older adults: a systematic review and meta-analysis
Source: Nutr J. 2018 Aug 27;17:82. doi: 10.1186/s12937-018-0387-1 (PMC6112122; doi:10.1186/s12937-018-0387-1)
Supplement: Supplementary file 1 — Table S1. Search strategy. Table S2. Detailed instructions of the modified NOS tool. Table S3. SoE grading system. (DOC 83 kb) [file 12937_2018_387_MOESM1_ESM.doc]

**Table S1 Search strategy**

| Search terms |
| --- |
| **PubMed (search date: 09/18/2017)** |
| #1 dairy [Title/Abstract]  #2 milk [Title/Abstract]  #3 yogurt [title/abstract] |
| #4 dementia [title/abstract]  #5 AD [title/abstract]  #6 Alzheimer* [title/abstract]  #7 aphronesia [title/abstract]  #8 cognitive* [title/abstract] |
| #9 “humans” [MeSH Terms]  #10 #1 OR #2 OR #3  #11 #4 OR #5 OR #6 OR #7 OR #8  #12 #9 AND #10 AND #11 |
| **CHINAHL (search date: 10/12/2017)** |
| S1 TI dairy OR AB dairy  S2 TI milk OR AB milk  S3 TI yogurt OR AB yogurt |
| S4 TI dementia OR AB dementia  S5 TI AD OR AB AD  S6 TI Alzheimer* OR AB Alzheimer*  S7 TI aphronesia OR AB aphronesia  S8 TI cognitive* OR AB cognitive* |
| S10 (S1 OR S2 OR S3) AND (S4 OR S5 OR S6 OR S7 OR S8); Limiters - Human |
| **EMBASE (search date: 10/12/2017)** |
| #1. dairy:ti,ab  #2. milk:ti,ab  #3. yogurt:ti,ab |
| #4. dementia:ti,ab  #5. ad:ti,ab  #6. alzheimer*:ti,ab  #7. aphronesia:ti,ab  #8. cognitive*:ti,ab |
| #9. 'human'/de  #10. dairy:ti,ab OR milk:ti,ab OR yogurt:ti,ab  #11. dementia:ti,ab OR ad:ti,ab OR alzheimer*:ti,ab OR aphronesia:ti,ab OR cognitive*:ti,ab  #12. 'human'/de AND (dairy:ti,ab OR milk:ti,ab OR yogurt:ti,ab) AND (dementia:ti,ab OR ad:ti,ab OR alzheimer*:ti,ab OR aphronesia:ti,ab OR cognitive*:ti,ab) |

**Table S2** Detailed instructions of the modified Newcastle-Ottawa Scale (NOS) scale^1^ for cohort studies

| ****SELECTION****  1. Representativeness of the exposed cohort:   - Is the exposed cohort appropriate and representative of the population of interest?   - High Risk: no (e.g., volunteers/self-recruitment, nurses); indication of subpopulation selective sampling in original cohort   - Low Risk: Yes, a truly representative or somewhat representative consecutive sample or random selection of the condition/population under study; they tried to do original sampling (complete) of original cohort, and original cohort large; no indication of selective sampling   - Unclear Risk: No description of the derivation of the cohort   2. Selection of the non-exposed cohort:   - Is the non-exposed cohort from the same community as the exposed cohort?   - High risk: no, drawn from a different source   - Low Risk: Drawn from the same community as the exposed cohort   - Unclear Risk: No description of the derivation of the non-exposed cohort   - (Note: Almost always low risk in cohort studies with nutrition (quintiles))   3.  Ascertainment of the Nutrient(s)’ Exposure:   - Did they use secure records (e.g., surgical records), structured interviews, or a validated instrument that can describe the exposure?   - High Risk: no, only written self-report or used a validated (or unvalidated) instrument on a specific day/in a way when usual intake is not described (e.g., only a single 24HR)   - Low Risk: Yes, they used a secure record, structured interview, or validated instrument that can describe the duration of exposure/intake over a given time period   - Unclear Risk: no description was given   4. Outcome of interest absent at baseline: (FOR NON-CONTINUOUS (EVENT) OUTCOMES ONLY; otherwise put N/A.)   - Did they demonstrate that outcome of interest was not present at the start of the study?   - High risk: no (Note: Unlikely answer to this question high risk on cohort studies)   - Low risk: yes (There is a statement of no history of disease or incidence at baseline)   - Unclear risk: they did not state whether it was present or absent at baseline, (e.g., missing baseline data on the population)   - NA: only continuous outcomes reported   ****COMPARABILITY****  5. Control for important confounders   - Did the study control for the required confounders that may influence the outcome(s) of interest?   - High risk: No, none/not all of them (They did not acknowledge these confounders when it was clear they were present, or they stated the confounders they adjusted for and these/some of these were missing from the list).   - Low risk: Yes, they adjusted for all these confounders in the analysis and/or exposed and non-exposed individuals were matched in the study design.   - Unclear risk: They did not report any confounders they controlled for (saying no differences between groups or that differences were not statistically significant are not sufficient for establishing comparability)   6. Adequate sample size and power   - Is the sample size adequate and is there sufficient power to detect a meaningful difference in the outcome of interest?   - High risk: Sample size was small and there was not enough power to test the outcome(s) of interest (*only an issue if NO significant finding)*   - Low risk: Sample size was adequate and there was enough power to test the outcome(s) of interest   - Unclear risk: Sample size and/or power were not addressed in the article.   ****OUTCOME****  7.  Outcome assessment   - How was the outcome(s) assessed? *(This question can be asked for each outcome of interest)*   - High risk: Only self-report (relied on subjective data as primary method to discern outcome status of participants)   - Low risk: independent, blind assessment, record linkage or laboratory measurements/biomarkers were used (the study used objective methods to discern the outcome status of participants)   - Unclear risk: no description was given/the study had limited reporting about assessment of outcomes   8. Completeness of cohort follow-up:   - Was there complete follow-up of all subjects?   - High risk: >20% of subjects were lost to follow-up; no description was given of those lost to follow-up; OR there was differential loss to follow-up between groups (losses should not be related to either the exposure or the outcome)   - Low risk: there was complete follow-up, with all subjects accounted for); <20% were lost to follow-up and thus unlikely to introduce bias; OR description was provided of those lost to follow-up   - Unclear risk: No follow-up rate was given   9. Selective outcome reporting (*similar to the RCT ROB heading) (do it for each outcome separately)*   - Is the study free of suggestion of selective outcome reporting?   - High risk: No, there were outcomes assessed in the methods/analyses sections of the paper that were not reported in the results section.   - Low Risk: Yes, all outcomes assessed in the methods/analyses sections of the paper were reported in the results section |
| --- |

^1^ Wells GA, Shea B, O'Connell D, Peterson J, Welch V, Losos M, Tugwell P. The Newcastle-Ottawa Scale (NOS) for assessing the quality of nonrandomised studies in meta-analyses. Ottawa: Ottawa Hospital Research Institute; 2011.

**Table S3** Strength of evidence grading system^1^

| **LEVEL A: Strong**  Clear evidence from at least 1 large, well-conducted, generalizable RCT that is adequately powered with a large effect size and is free of bias or other concerns  **OR**  Clear evidence from multiple RCTs or many controlled trials that may have few limitations related to bias, measurement imprecision, inconsistent results, or other concerns (i.e., incorporated quality ratings)  **Level B: Moderate**  Evidence obtained from multiple, well-designed, conducted, and controlled prospective cohort studies that have used adequate and relevant measurements and that gave similar results from different populations  **OR**  Evidence obtained from a well-conducted meta-analysis of cohort studies or prospective cohort studies from different populations  **Level C: Limited**  Evidence obtained from multiple prospective cohort studies from diverse populations that have limitations related to bias, measurement imprecision, or inconsistent results or have other concerns  **OR**  Evidence from only 1 well-designed prospective study with few limitations  **Level D: Inadequate**  Evidence from studies that have 1 or more major methodologic flaws or many minor methodologic flaws that result in low confidence in the effect estimate  **OR**  Insufficient data to support a hypothesis  **Level E- Expert Consensus or Clinical Experience**  This is a separate category for recommendations in which there is as yet no evidence from clinical trials, in which clinical trials may be impractical, or in which there is conflicting evidence  **Level NA**  No studies have been done pertaining to question being addressed- indicates research gap/area for future research |
| --- |

^1^ The grading system is based on an article from the American Diabetes Association (ADA) and an article published by ASN in the AJCN (17, 18). NA, not applicable; RCT, randomized controlled trial; SOE, strength of evidence.
